# High-Throughput Sequencing Analysis of Microbiota and Enzyme Activities in *Xiaoqu* from Seven Provinces in Southern China

**DOI:** 10.4014/jmb.2405.05029

**Published:** 2024-09-09

**Authors:** Weiwei Dong, Jingjing Zhang, Menglin Zou, Liang Chen, Liping Zhu, Long Zhang, Gang Zhang, Jie Tang, Qiang Yang, Yuanliang Hu, Shenxi Chen

**Affiliations:** 1 Hubei key Laboratory of Quality and Safety of Traditional Chinese Medicine Health Food, Jing Brand Co., Ltd., Daye, Hubei 435100, P.R. China; 2 Hubei Key Laboratory of Edible Wild Plants Conservation and Utilization, College of Life Sciences, Hubei Normal University, Huangshi 435002, P.R. China

**Keywords:** *Xiaoqu*, microbial community, *Bacillus*, lactic acid bacteria, yeasts, molds

## Abstract

*Xiaoqu*, a pivotal starter in baijiu fermentation, provides the most microflora and enzymes to initiate and maintain baijiu brewing. This study aims to explore the differences in microbiota and enzyme activities among *Xiaoqu* samples from seven provinces in southern China using high-throughput sequencing, plate isolation, and activity detection. The analyses revealed significant differences in bacterial and fungal communities across the samples. A total of 22 bacterial species and 17 target fungal species were isolated and identified. Predominant bacteria included *Bacillus* (*Bacillus subtilis*) and lactic acid bacteria (LABs), while the fungal communities were primarily composed of yeasts (*Saccharomyces cerevisiae*) and various molds. The activities of α-amylase and glucoamylase varied significantly among the samples, and samples from HN1 and GZ2 exhibited the highest activities. Correlation analyses highlighted the pivotal role of LABs in maintaining acidity and the importance of molds and yeasts in the saccharification and fermentation processes. These findings shed light on the microbial composition and diversity of *Xiaoqu* and the critical role of microbes in baijiu production. Moreover, they suggested potential microbial resources for developing artificial *Xiaoqu* via synthetic microbial community in the future, enhancing baijiu fermentation efficiency and overall product quality.

## Introduction

Baijiu, one of the six major distilled spirits globally, is renowned for its unique brewing technique and deep cultural heritage, earning a beloved status worldwide [1]. Central to baijiu production is *Jiuqu*, the fermentation starter, that introduces essential microflora, enzymes, and some aromatic precursors for baijiu brewing [2, 3]. Among *Jiuqu*, *Daqu* and *Xiaoqu* are the most widely utilized, with differences in size - *Daqu* being larger (1.5-4.5 kg) and *Xiaoqu* smaller (10-100 g), to brew distinct types of baijiu [4, 5]. For instance, strong-flavor and sauce-flavor baijiu are primarily produced using *Daqu*, while southern light-flavor and rice-flavor baijiu often rely on *Xiaoqu* [6-8]. The brewing processes of baijiu applied *Xiaoqu* are characterized by the use of a smaller amount of *Jiuqu* (0.8%-1.5%), a shorter fermentation period (7-30 days), and a higher liquor yield (up to 60%), making it increasingly favored by consumers, especially in southern China [9-11].

Microbial diversity within *Xiaoqu* plays a crucial role in the complex fermentation processes of baijiu, directly impacting the flavor, quality, and stability of the final product [3, 5]. The core of baijiu brewing is the metabolism of various microbes during fermentation, where macromolecular substrates in raw material are transformed into ethanol and flavor substances [12, 13]. Currently, the study of the microbial diversity of *Xiaoqu* is a burgeoning field. Cai *et al*. investigated the microbial community of 8 *Xiaoqu* samples from southern China, identifying 10 genera of yeasts and molds, along with 11 genera of bacteria [14]. Subsequently, *Xiaoqu* samples from 4 provinces in southern China were analyzed, revealing significant differences in the diversity of microbial community among samples [15]. Zhao’s study examined the microbial community of three typical traditional *Xiaoqu* from the Guizhou province, and the results showed that *Lactobacillus*, *Bacillus*, *Acinetobacter*, *Leuconostoc*, and *Weissella* were the dominant bacteria genera, while the predominant fungal genera belonged to *Aspergillus*, *Saccharomyces*, *Pichia*, *Rhizopus*, and *Phycomyces* [16]. However, current researches on the microbial composition of *Xiaoqu* mainly focuses on specific regions or types of samples, lacking comprehensive and systematic analyses across a broader geographic scope in China. Furthermore, the sequencing methods used in these studies are limited to identification at the genus level, failing to provide precise taxonomic status. In addition, the isolation of cultivable microbes from *Xiaoqu* is crucial for identifying and providing potential core functional microbes because the microbes within *Xiaoqu* emerge as the cornerstone of baijiu fermentation. Isolation and identification of functional microbes will lay a solid foundation for the production of artificial *Xiaoqu* from the aspect of synthetic microbial community in the future, aiming to address the issue of *Xiaoqu* instability caused by uncontrollable spontaneous fermentation in traditional production processes of *Xiaoqu* [17, 18].

Given these gaps, this study seeks to systematically compare the microbial composition, cultivable microbes, and enzyme activities of *Xiaoqu* samples from seven provinces in southern China. By employing third-generation high-throughput sequencing (HTS) alongside a systematic evaluation of cultivable microbes and enzyme activities, this research aims to deepen our understanding of the variations in brewing characteristics and microbiota of *Xiaoqu*. This not only contributes to a deeper visualization of the brewing processes but also provides a scientific basis by offering valuable microbial resources for improving the quality and competitiveness of *Xiaoqu* baijiu products, promoting the healthy development of *Xiaoqu* baijiu industry in southern China.

## Materials and Methods

### *Xiaoqu* Sampling

All the produced *Xiaoqu* samples passed quality examination by the production factory, and then samples from seven provinces, consisting of Sichuan, Hubei, Hunan, Jiangxi, Guizhou, Yunnan, and Guangxi, in southern China were collected. Two distinct types of *Xiaoqu* samples from each province were gathered with duplications, resulting in a total of 28 samples (Table 1). These samples were respectively crushed, packaged, and promptly transported to the laboratory under storage of 4°C for subsequent analyses, including assessment of physicochemical properties, enzyme activities, cultivable microbes, and microbial community.

### Physicochemical Properties

The physicochemical properties and enzyme activities of all the *Xiaoqu* samples were detected based on previous studies [19, 20]. Moisture content was assessed via weighing and taking 4 g of *Xiaoqu* sample into an oven for desiccation (overnight) until a constant weight, and calculating the ratio of weight loss. The acidity of *Xiaoqu* sample was determined by the method of acid-base titration. Namely, 5 g of *Xiaoqu* sample was added into 20 ml ddH_2_O, and the mixture was then vibrated under the condition of 25°C and 180 rpm for 1 h to acquire acids extraction. Afterwards, the 0.05 M NaOH was applied to neutralize the acids, and the amount of used NaOH was record to calculate the acidity.

### Enzyme Activities

The crude enzyme solution was extracted from *Xiaoqu* sample before determining enzyme activities. A total of 5g *Xiaoqu* sample was added into 45 ml ddH_2_O and incubated at 25°C and 180 rpm with a duration of 1 h for extraction. Subsequently, the extracting mixture was filtered for measuring α-amylase and glucoamylase. The α-amylase activity was tested by using filtrate to react with starch solution, and the residual starch was determined by iodine-starch colorimetry at 660 nm. The glucoamylase activity was evaluated by assessing the amount of glucose produced from starch degradation by glucoamylase, and the released glucose was quantified by DNS (3,5-dinitrosalicylic acid) method according to the standard glucose solution. The saccharifying power of *Xiaoqu* was measured based on the national standard (QB/T 4257-2011) according to Xiao’s study [20]. One unit of saccharifying power of *Xiaoqu* was defined as the number of milligrams of soluble starch converted into glucose by 1 g of *Xiaoqu* under the conditions of 35°C and pH 4.6 within 1h. In the same way, the resultant glucose was determined by DNS method according to standard glucose solution.

### Isolation and Identification of Cultivable Microbes

The cultivable microbes in *Xiaoqu* were isolated using the traditional dilution and plating method, with modifications based on our previous studies [19, 21]. The target microbes for isolation and identification included *Bacillus*, lactic acid bacteria (LABs), yeasts, and molds. Firstly, 10 g of *Xiaoqu* sample was mixed with 90 ml sterile H_2_O, and shaken at 160 rpm for 30 min. Subsequently, the supernatant of the mixture was acquired after 10 min of static settling and used for gradient dilution until reaching an appropriate dilution. Finally, 100 μl of diluent was plated onto different plates to isolate the target microbes. Specifically, LB agar, MRS agar (with nystatin), YPD agar (with ampicillin and 0.33% acetic acid), and Potato Dextrose Agar (PDA) (with ampicillin) were used to isolate *Bacillus*, LABs, yeasts, and molds, respectively. Incubation temperatures were set at 37°C for LB agar and MRS agar, 28°C for YPD agar, and 25°C for PDA to facilitate the growth of the respective microbes.

Once single colonies of microbes appeared and grew to an appropriate size on each plate, they were subjected to morphological observation and microscopic examination. The number of different microbes was enumerated, suspected candidates were transferred to new plates using streak inoculation. The newly grown single colonies of related microbes were identified through colony PCR with the help of lysis buffer (5% Chelex-100 resin, Bio-Rad, USA) to facilitate the release of microbial DNA and improve the success rate of colony PCR. Specific primer pairs together with Taq DNA polymerase (2 × Rapid Taq Master Mix, Vazyme, China) were used for amplifying the target DNA of *Bacillus*, LABs, yeasts, and molds. Here, primer pairs *gyrA*-F (CAGTCAGGAAATGCGTACGTCCTT) and *gyrA*-R (CAAGGTAATGCTCCAGGCATTGCT) along with procedures (95°C 15 s, 51°C 15 s, 72°C 30 s, for 30 cycles) were used for identifying *Bacillus* [22], 27F (AGRGTTYGATYMTGGCTCAG) and 1492R (RGYTACCTTGTTACGACTT) with procedures (95°C 15 s, 54.5°C 15 s, 72°C 45 s, for 30 cycles) for LABs, NL1 (GCATATCAATAAGCGGAGGAAAAG) and NL4 (GGTCCGTGTTTCAAGACGG) with procedures (95°C 15 s, 52°C 15 s, 72°C 30 s, for 30 cycles) for yeasts, and ITS1 (TCCGTAGGTGAACCTGCGG) and ITS4 (TCCTCCGCTTATTGATATGC) together with procedures (95°C 15 s, 52°C 15 s, 72°C 30 s, for 30 cycles) for molds. Afterwards, the PCR products were sequenced by Tsingke Biotech Co., Ltd. (China) based on Sanger sequencing. The resulting sequences were subjected to BLAST analysis in NCBI (https://blast.ncbi.nlm.nih.gov), EzBioCloud (https://www.ezbiocloud.net) and Yeasts (https://theyeasts.org) to search for closely related sequences with species names. Finally, all these close sequences, together with the target sequence, were used to construct a neighbor-joining phylogenetic tree (MEGA 7.0) to identify the taxonomic status of the candidate microbes.

### Microbial Community Analysis based on Amplicon Sequencing

To accurately analyze the microbial community at species level, the third generation HTS technology-based amplicon sequencing was applied here. Initially, total DNA extraction from *Xiaoqu* samples was performed following the instructions of E.Z.N.A. Soil DNA Kit (Omega Bio-Tek, USA). Subsequently, the concentration and quality of extracted total DNA were accessed using Nanodrop and analysis of agarose gel electrophoresis, respectively. The eligible DNA of each *Xiaoqu* sample was further used as a template for library construction and third generation HTS according to our previous study [19]. Specifically, the full-length 16S rDNA and ITS region were amplified by using Q5 high-fidelity DNA polymerase (NEB, USA) with primer pairs, consisting of 27F (AGRGTTTGATYNTGGCTCAG) and 1492R (TASGGHTACCTTGTTASGACTT), and ITS1-F (CTTGGT CATTTAGAGGAAGTAA) and ITS4 (TCCTCCGCTTATTGATATGC), respectively [23, 24]. Finally, the purified PCR library was sequenced via PacBio Sequel II platform (Illumina NovaSeq 6000).

Post-sequencing data underwent bioinformatics analysis mainly using QIIME2 (2019.4). The original reads were filtered, denoised, and chimeras removed, along with discarding low-quality and contaminated reads (*e.g.*, mitochondria/chloroplasts) by Vsearch (v2.13.4) and cutadapt (v2.3). The obtained high-quality sequences were then clustered (at a 97% threshold) to generate operational taxonomic units (OTUs) by Vsearch (v2.13.4). The representative OTU sequences were BLAST and annotated via databases UNITE and Silva for fungi and bacteria, respectively, via QIIME2 (2019.4). The resultant annotated information was then utilized to calculate relative abundance at different taxonomic levels (phylum, genus, and species) via QIIME2 (2019.4). Finally, the corresponding diversity index, principal component analysis (PCA), clustering barplot, and Pearson correlation analysis were conducted via Personalbio platform (https://www.genescloud.cn).

### Statistical Analysis

Statistical analyses in this study involved data processing with Excel (version 2019) and RStudio (version 2023.09.0), and the determination of significance was conducted using Origin (version 9.0) by the One-Way ANOVA analysis (*p* < 0.05). The Bray-Curtis distance was used in PCA with confidence at 0.95, and the confidence interval of R in Pearson correlation analysis was set as 0.6.

### Data Availability

The raw data generated from HTS has been uploaded and preserved in NCBI under the BioProject accession number PRJNA1106182 and PRJNA1106188.

## Results and Discussion

### Physicochemical Properties of *Xiaoqu*

**Fig. 1:**
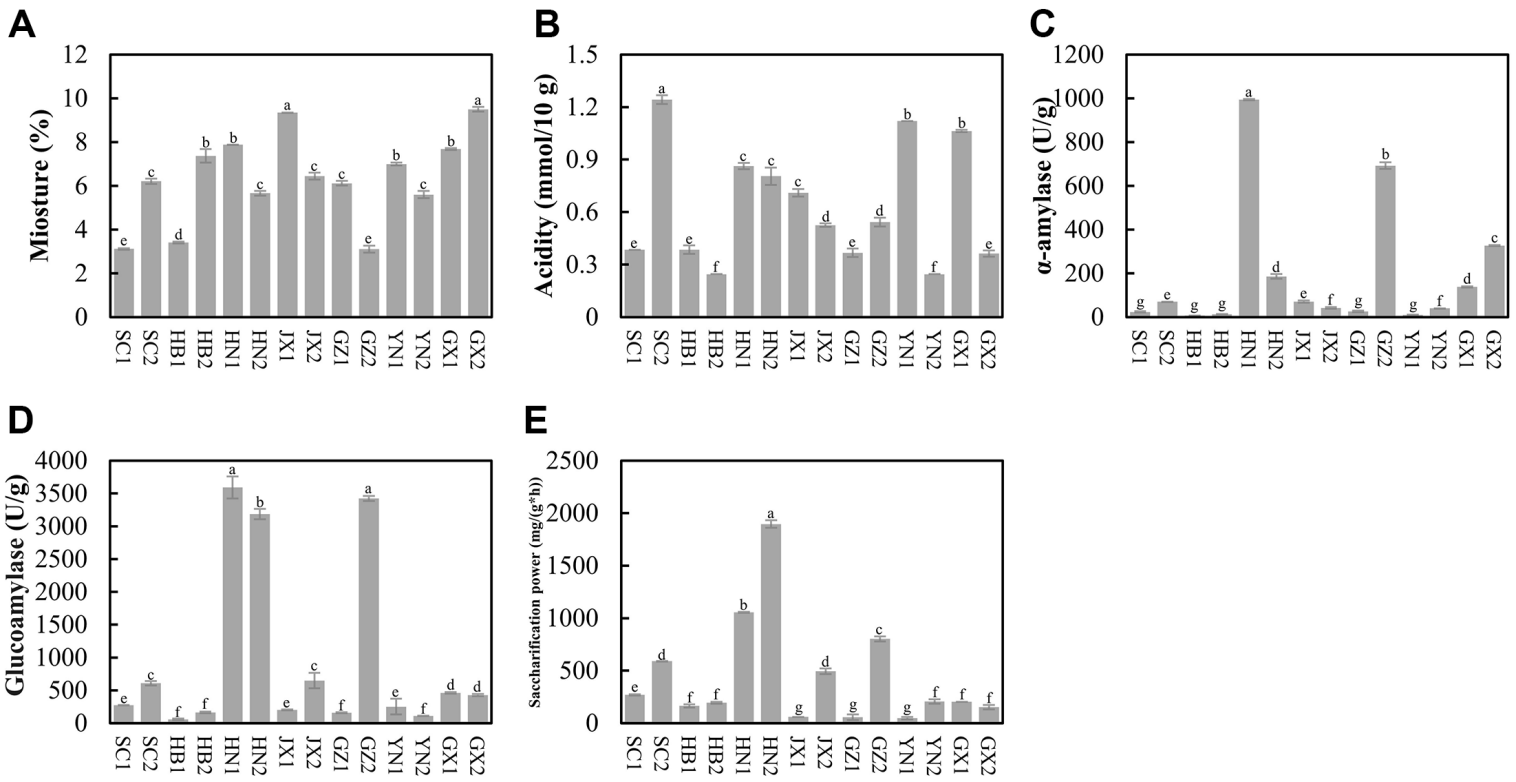
The physicochemical properties and enzyme activities of *Xiaoqu* from seven provinces in southern China, including moisture (A) acidity (B) α-amylase (C) glucoamylase (D) and saccharification power (D). The One-Way ANOVA was performed to evaluate the significant differences (*p* < 0.05). The same letter represented no significant differences between the chosen *Xiaoqu* samples, while the different letters revealed significant differences between the *Xiaoqu* samples.

The physicochemical properties and enzyme activities of all *Xiaoqu* samples from seven provinces in southern China were shown in Fig. 1. The moisture content varied from 3.10% to 9.50% (Fig. 1A), aligning with findings from previous study on *Xiaoqu* from Guizhou province [16]. It was reported that the microbial activities and enzyme activities in *Jiuqu* could be better preserved when the water content is less than 10% [3, 25], indicating that the moisture of all *Xiaoqu* samples falls within an appropriate range. Notably, GX2 (9.50%) and JX1 (9.34%) *Xiaoqu* gained significantly higher moisture content, while GZ2 (3.11%) and SC1 (3.10%) *Xiaoqu* exhibited relatively lower moisture content (Fig. 1A). Most of the *Xiaoqu* maintained moisture content between 5.60% and 7.88% (Fig. 1A), slightly lower than that of high-temperature *Daqu* (9%-10%) from Guizhou province [26]. This difference in moisture might be caused by the sizes of the two types of *Jiuqu*. Despite minor discrepancy in moisture, both *Xiaoqu* and *Daqu* outlined low water content below 10%, underscoring the importance of low humidity for maintaining microbial and enzyme activities in *Jiuqu* [27]. Regarding acidity, *Xiaoqu* samples fluctuated ranging from 0.24 mmol/10 g to 1.24 mmol/10 g, with SC2 (1.24 mmol/10 g), YN1 (1.12 mmol/10 g), and GX1 (1.06 mmol/10 g) showing the significantly highest acidity (Fig. 1B). *Xiaoqu* of HN1, HN2, JX1, JX2, and GZ2 ranged from 0.50 mmol/10 g to 1.00 mmol/10 g, with the rest below 0.50 mmol/10 g (Fig.1B). The acidity levels observed in our *Xiaoqu* samples were consistent with those reported in previous studies [16, 28]. Acidity serves as a crucial indicator for *Jiuqu* quality assessment, reflecting the metabolic activity of acid-producing microbes, particularly the LABs, which play a vital role in the initial stages of *baijiu* brewing by rapidly proliferating and inhibiting harmful microbes, thereby ensuring fermentation safety [29, 30].

### Enzyme Activities of *Xiaoqu*

Enzyme activities are crucial indicators of *Xiaoqu* quality, as they reflect its saccharification ability-rapidly breaking down starch into reducing sugars, providing the carbon source for microbial growth and initiate baijiu fermentation [3, 31]. Moreover, these fast-growing microbes can produce flavor compounds, thereby impacting the final quality of baijiu [32]. The results of enzyme activities in *Xiaoqu* from seven provinces in southern China were depicted in Fig. 1. Significant variations were observed in α-amylase and glucoamylase activities across different regions (Fig. 1C and 1D). Only three *Xiaoqu* samples exhibited α-amylase activity exceeding 200 U/g—HN1 (994.04 U/g), GZ2 (692.06 U/g), and GZ2 (326.98 U/g); two samples ranged between 100 U/g and 200 U/g—HN2 (185.55 U/g) and GX1 (138.16 U/g); while the remaining *Xiaoqu* samples showed α-amylase activity below 100 U/g (Fig. 1C). Overall, *Xiaoqu* displayed higher α-amylase activity compared to *Daqu*, with values typically below 40 [19]. As for glucoamylase activity, HN1, GZ2, and HN2 exhibited the significantly highest activity, exceeding 3000 U/g, while other *Xiaoqu* samples showed lower activity below 1000 U/g (Fig. 1D). Specifically, HB1 (59.36 U/g), YN2 (111.73 U/g), GZ1 (159.08 U/g), and HB2 (163.42 U/g) displayed the lowest glucoamylase activity. Although the glucoamylase activity of *Xiaoqu* samples differed, it still maintained higher activity than that of high-temperature *Daqu* samples [26]. The saccharification power of *Xiaoqu* samples generally mirrored glucoamylase activity results. HN2, HN1, and GZ2 showed the significantly highest saccharification power, with values of 1896.01 mg/(g·h), 1056.60 mg/(g·h), and 804.05 mg/(g·h), respectively, which was as high as Zhao’s study [16]. Conversely, the samples with the lowest saccharification power were mainly concentrated in *Xiaoqu* JX1, GZ1, and YN1, all exhibiting values below 100 mg/(g·h) (Fig. 1E). Despite some samples showing extremely low saccharification power, they have been utilized for years by local liquor companies, indicating that the most important of *Xiaoqu* is the microbes it contains.

### Isolation and Identification of Cultivable Microbes from *Xiaoqu*

**Fig. 2:**
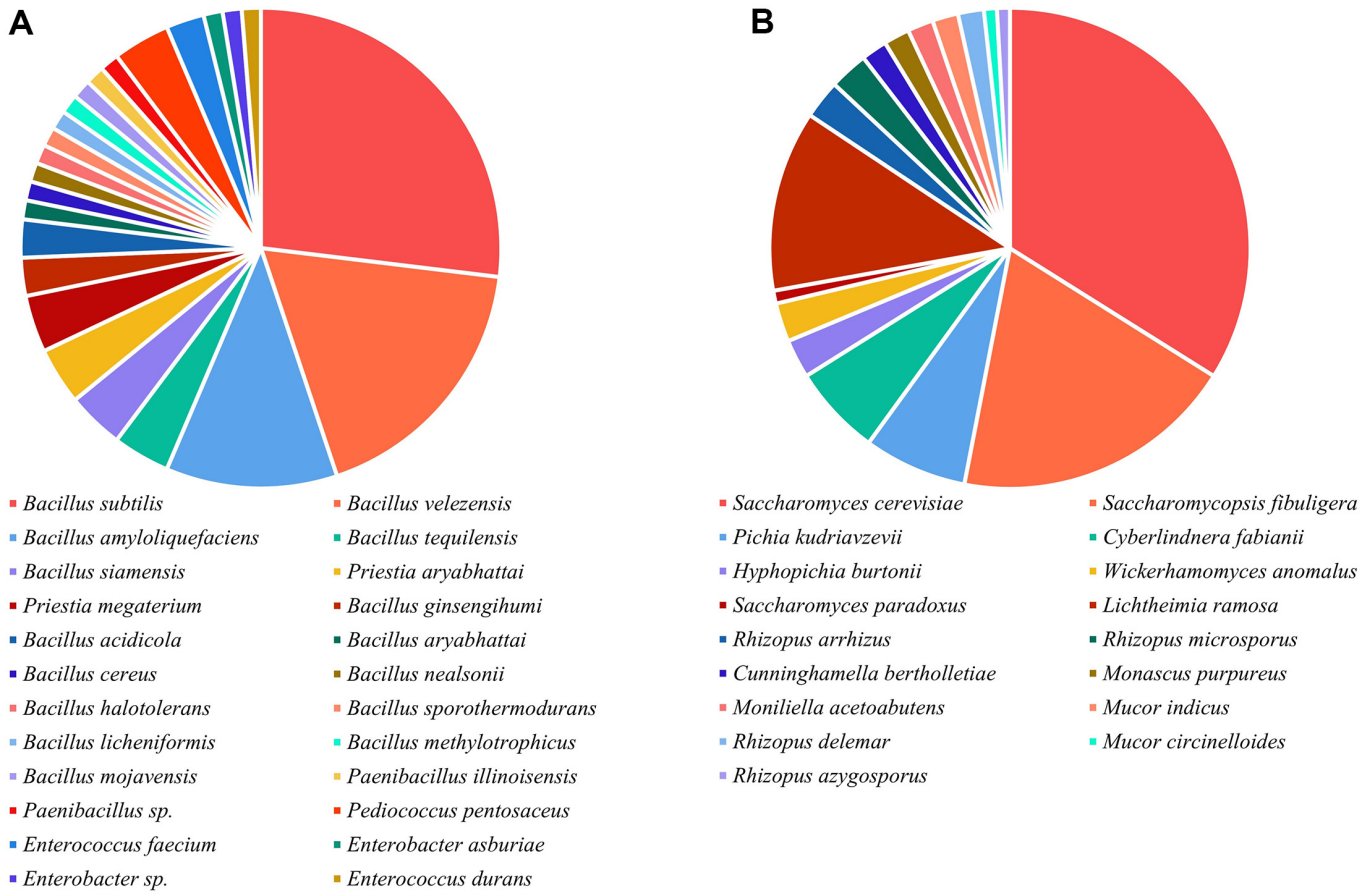
The culturable microbes of *Xiaoqu* from seven provinces in southern China, including bacteria (A) and fungi (B).

Isolation, identification, and enumeration of cultivable microbes from *Xiaoqu* revealed a diverse microbial community comprising 22 bacterial species (Fig. S1), including *Bacillus* and LABs, and 17 target fungal species, encompassing yeasts and molds (Fig. 2). *Bacillus* were the most abundant bacteria, with *Bacillus subtilis* (26.92%), *Bacillus velezensis* (17.95%), *Bacillus amyloliquefaciens* (11.54%), *Bacillus tequilensis* (3.85%), and *Bacillus siamensis* (3.85%) ranking among the top 5 species, and *B. subtilis* was the most dominant species. *Bacillus* are commonly found in *Jiuqu*, and are known to secrete various hydrolases, such as amylases, proteases, and lipases, promoting the smooth fermentation processes, and improve flavor and quality of baijiu [10, 33, 34]. Notably, co-inoculation of *B. subtilis* and *B. velezensis* has been shown to impact *Jiuqu* enzymes and microbiome, improving flavor quality [35], while Yang’s study found that the inoculation of *B. subtilis* modified the microbiome of *Jiuqu*, especially enhancing interactions between bacteria and fungi [36]. Thus, isolating *Bacillus* resources in *Jiuqu* could be the basis for modern *Jiuqu* production in future. Besides *Bacillus*, two species of *Paenibacillus*, *P. illinoisensis* and *Paenibacillus* sp., were isolated (Fig. 2A), which were reported rarely in *Jiuqu*. LABs were predominantly represented by species from the genera *Pediococcus*, *Enterococcus*, and *Enterobacter*, with *Pediococcus pentosaceus* being the most abundant (3.85%) (Fig. 2A). Interestingly, cocci-shaped LABs were more prevalent (7.69%) than bacilli-shaped LABs (2.56%), suggesting greater resistance among cocci-shaped LABs. Notably, no species from the genus *Lactobacillus* were isolated, indicating potential unsuitability of *Lactobacillus* for *Xiaoqu* production, particularly during prolonged storage under low-moisture conditions, leading to their demise [37]. Additionally, it was found that the isolated LABs mainly originated from *Xiaoqu* samples SC2, YN2, GX1, HN2, and JX1, which had relatively higher level of acidity (Fig. 1B), suggesting that the acidity of *Xiaoqu* originates from the metabolite of lactic acid by these LABs [10].

In terms of fungi, 7 yeast species and 10 mold species were isolated and identified (Fig. 2B). The top three yeast species included *Saccharomyces cerevisiae* (33.91%), *Saccharomycopsis fibuligera* (19.13%), and *Pichia kudriavzevii* (6.96%), while *Lichtheimia ramosa* (12.17%), *Rhizopus arrhizus* (2.61%), and *Rhizopus microsporus* (2.61%) constituted the top three mold species (Fig. 2B). These yeast species are essential for baijiu brewing [34], with *S. cerevisiae* is known for its high ethanol production [9], *S. fibuligera* capable of producing ethanol, α-amylase, β-glucosidase and esters efficiently [38, 39], and *P. kudriavzevii* contributing to the formation of flavor compounds, including isoamyl alcohol and ethyl acetate [40]. Among the isolated molds, *R. arrhizus* *R. microsporus*, and *L. ramosa* are frequently found in *Xiaoqu* [17, 41]. *R. arrhizus* and *R. microspores* were famous for secreting α-amylase, β-glucosidase, attributing to saccharification power of *Jiuqu*. *L. ramosa* was also found in the starter used for traditional rice wine in Korea [42], moreover, *L. ramose* had the ability of producing β-glucosidase and xylanase [43]. Further research is warranted to explore the characteristics and specific functions of these strains to uncover more functional microbial resources for baijiu brewing.

### Microbial Diversity of *Xiaoqu*

Microbial diversity analysis of *Xiaoqu* revealed valuable insights into the evenness and richness of bacterial and fungal communities. Following stringent data processing, a total of 7,487,454 high-quality bacterial reads (an average of 267,409 reads per sample) and 2,497,879 high-quality fungal reads (an average of 89,209 reads per sample) were obtained, with robust sequence coverages exceeding 0.999 across all samples, ensuring the reliability of the amplicon sequencing results. These reads were annotated into diverse taxonomic groups, comprising 5 phyla, 193 genera, and 431 species for bacteria, and 4 phyla, 161 genera, and 519 species for fungi. To assess α-diversity, Chao1 richness and Shannon index were calculated.

Regarding bacterial Chao1 richness, values ranged from 9.00 to 88.38, with GX1 the lowest (9.00) and JX1 the highest (88.38) (Table 2). Most *Xiaoqu* samples, including SC1, SC2, HN1, HN2, JX2, GZ1, GZ2, and YN2, displayed Chao1 richness between 40 and 60, indicating comparable bacterial richness among these *Xiaoqu* samples. However, the Chao1 richness of GX1, HB1, HB2, and YN1 were all below 30, much lower than those of other *Xiaoqu* samples, suggesting lower bacterial diversity of these samples. Fungal Chao1 richness ranged from 26.71 to 180.75, with GX1 again demonstrating the lowest (16.71) and HN2 the highest (180.75). Despite some variation, fungal Chao1 richness across different provinces generally fell between 60 and 150, except for GX1, GX2, JX1, and HN2. Notably, fungal Chao1 richness exceeded bacterial richness by 1.2 to 4.0 times, underscoring the predominance of fungi in *Xiaoqu*. For Shannon indices, they mirrored the trends observed in Chao1 richness (Table 2). The Shannon indices of bacterial communities ranged from 0.26 to 2.65, with GX1 displaying the lowest (0.26) and GX2 the highest (2.65). For most provincial *Xiaoqu*, the Shannon indices of bacterial communities showed little variation, ranging from 0.8 to 2.2. Similarly, the Shannon indices of fungal communities ranged from 0.86 to 2.19, with GX1 exhibiting the lowest (0.86) and HB1 the highest (2.19). Overall, the *Xiaoqu* samples from GX1 had the lowest α-diversity, potentially due to unique production processes. Additionally, fungi consistently exhibited higher diversity than bacteria in the same *Xiaoqu* sample, emphasizing their pivotal role in fermentation.

**Fig. 3:**
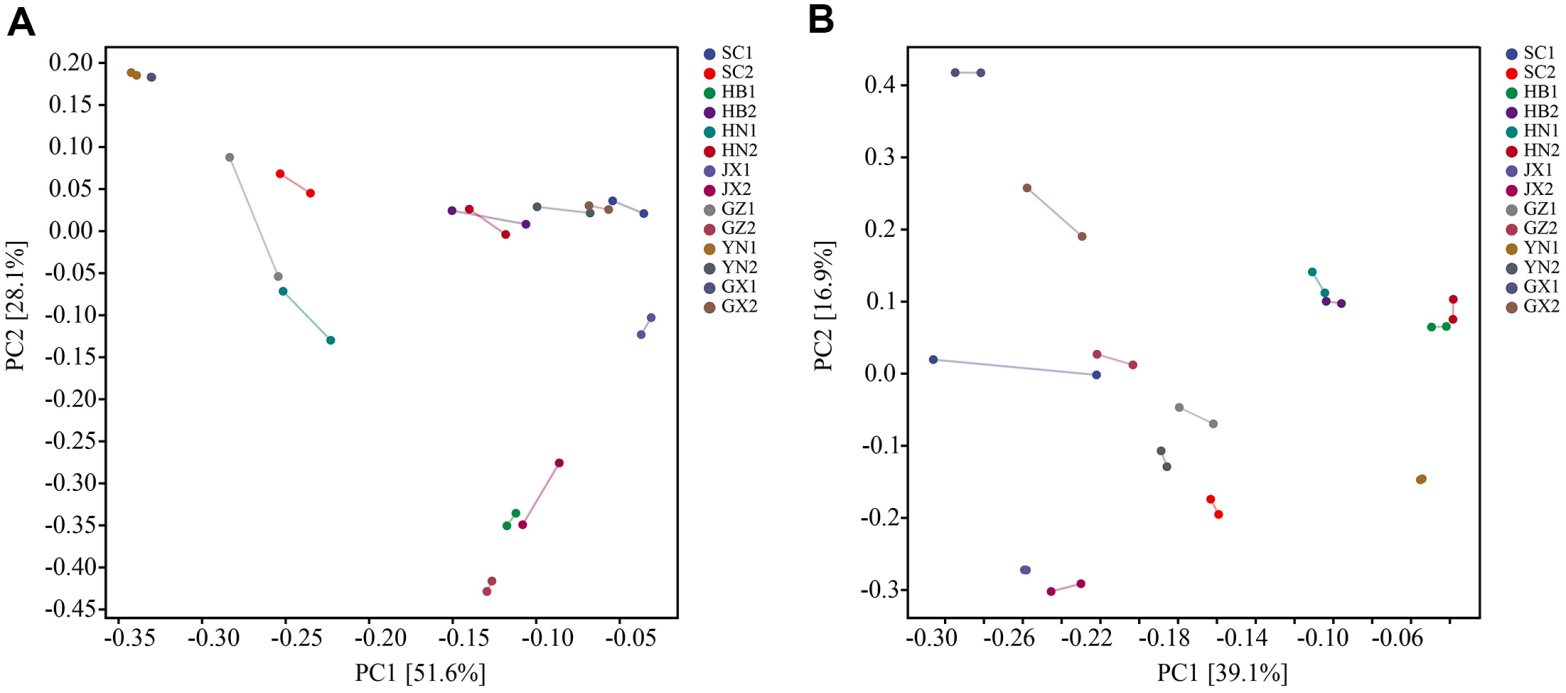
The microbial community diversity of *Xiaoqu* from seven provinces in southern China, bacterial community β-diversity (A) and fungal community β-diversity (B). The Bray-Curtis distance (R2 = 0.8896. *p* = 0.0001) was used in β-diversity analysis with confidence at 0.95.

The β-diversity analysis through PCA elucidated the variation among *Xiaoqu* samples from different provinces (Fig. 3). Results indicate that the cumulative explained variance of the bacterial PCA plot's horizontal and vertical axes covers 79.7% (Fig. 3A), while that of the fungal PCA plot reaches 56.0% (Fig. 3B). Samples from the same manufacturer within one province exhibited coherence, yet significant differences were observed among manufacturers within the same province. Moreover, no discernible correlation was found among samples from different provinces (Fig. 3), possibly attributable to geographical barriers and the considerable distances separating sampling locations (over 80 km from each other).

### Microbial Community Composition of *Xiaoqu*

**Fig. 4:**
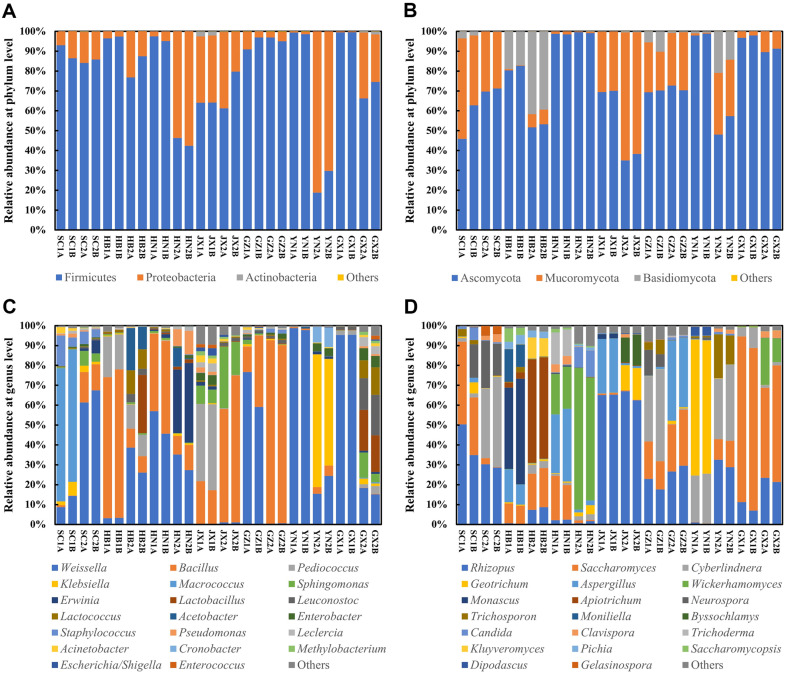
The microbial community of *Xiaoqu* from seven provinces in southern China, including bacterial phylum level (A) fungal phylum level (B) bacterial genus level (C) and fungal genus level (D).

Although the above results indicated differences in microbial diversity among *Xiaoqu* samples from seven provinces in southern China, the specific microbial composition of *Xiaoqu* samples was still unknown. By analyzing the composition of microbial community in *Xiaoqu* at different taxonomic levels, we can understand the specific microbial differences. At the phylum level, the dominant bacterial phyla were Firmicutes (18.78%-99.58%, average 79.41%), Proteobacteria (0.42%-81.22%, average 20.25%), and Actinobacteria (0-2.52%, average 0.27%) (Fig. 4A), which was consistent with previous study [16]. Proteobacteria became the most dominant phylum in YN2 (75.75%) and HN2 (55.62%), while all other *Xiaoqu* samples were mostly dominated by Firmicutes (Fig. 4A). The dominant fungal phyla were Ascomycota (34.98%-99.52%, average 74.50%), Mucoromycota (0.35%-64.48%, average 19.10%), and Basidiomycota (0-41.76%, average 6.40%) (Fig. 4B), coincided with previous study [16]. Ascomycota predominated in most *Xiaoqu* samples, except for JX2, where Mucoromycota (62.89%) was dominant (Fig. 4B).

At the genus level, the top 20 dominant genera of bacteria were shown in Fig. 4C, where the top 5 genera in abundance included *Weissella* (average 35.30%), *Bacillus* (average 25.41%), *Pediococcus* (average 5.50%), *Klebsiella* (average 5.15%), and *Macrococcus* (average 4.81%) (Fig. 4C). However, *Staphylococcus*, which was previously reported as the most dominant genus by Tang’s study (average 37.98%) [15], exhibited relatively low abundance (1.30%) in this study (Fig. 4C). This discrepancy may be due to differences in sample collection methods or regional environmental factors. To further elucidate the consistency among all *Xiaoqu* samples, clustering analysis based on bacterial genus level revealed eight distinct clusters (Fig. S2A). *Xiaoqu* SC2, GZ1, and HN1 had similar bacterial compositions forming Cluster 1, while YN1 and GX1 formed Cluster 2 (Fig. S2A). Samples in Cluster 1 and Cluster 2 were dominated by *Weissella*, a common LABs genus capable of producing lactic acid to increase sample acidity [10], which may be the reason for the highest acidity of *Xiaoqu* SC2, YN1, and GX1 (Fig. 1B). Additionally, HB2 and GX2 formed Cluster 4; HB1, GZ2, and JX2 formed Cluster 6; and the remaining *Xiaoqu* samples clustered separately (Fig. S2A). Thereinto, Cluster 5 was dominated by *Klebsiella*, Cluster 6 was dominated by *Bacillus*, and Cluster 8 was dominated by *Macrococcus* (Fig. S2A). *Klebsiella* were also reported in other *Xiaoqu* samples, they could synthesize acetoin, which is the precursor of flavor substance tetramethylpyrazine [6, 16]. *Bacillus*, common bacteria in *Xiaoqu*, are known to produce various enzymes, enhancing the fermentation processes and baijiu quality [10, 33, 34]. The higher abundance of *Bacillus* in samples from Hubei province contrasted with previous study [15]. This discrepancy may be due to differences in sample collection methods or regional environmental factors. *Macrococcus*, known for its contribution to aroma and flavor in cheeses [44], was rarely reported in baijiu brewing. This highlights the potential role of *Macrococcus* in shaping the sensory characteristics of *Xiaoqu* baijiu. The presence of diverse microbial genera underscores the complexity of *Xiaoqu* microbial community and its potential impact on baijiu fermentation and flavor development.

Fungi showed similar patterns at the genus level compared to bacteria, with significant differences in the fungal composition at the genus level among different *Xiaoqu* samples (Fig. 4D). The top 5 dominant fungal genera were *Rhizopus* (average 24.73%), *Saccharomyces* (average 21.95%), *Cyberlindnera* (average 12.45%), *Wickerhamomyces* (average 9.77%), and *Aspergillus* (average 9.45%) (Fig. 4D). These findings diverged from previous study, which identified *Aspergillus*, *Saccharomyces*, *Pichia*, *Rhizopus*, and *Phycomyces* as the predominant fungi in *Xiaoqu* [16]. Similarly, clustering analysis further delineated eight distinct clusters, each characterized by specific fungal compositions (Fig. S2B). Cluster 1 were mainly dominated by *Rhizopus* and *Saccharomyces*; Cluster 3 by *Rhizopus* and *Cyberlindnera*; Cluster 4 by *Rhizopus*; Cluster 5 by *Apiotrichum*; Cluster 6 by *Monascus*; Cluster 7 by *Geotrichum*; and Cluster 8 by *Wickerhamonyces* (Fig. S2B). Overall, fungi in *Xiaoqu* primarily consisted of molds and yeasts [15, 41], playing a critical role in the saccharification and fermentation of *Xiaoqu* baijiu.

**Fig. 5:**
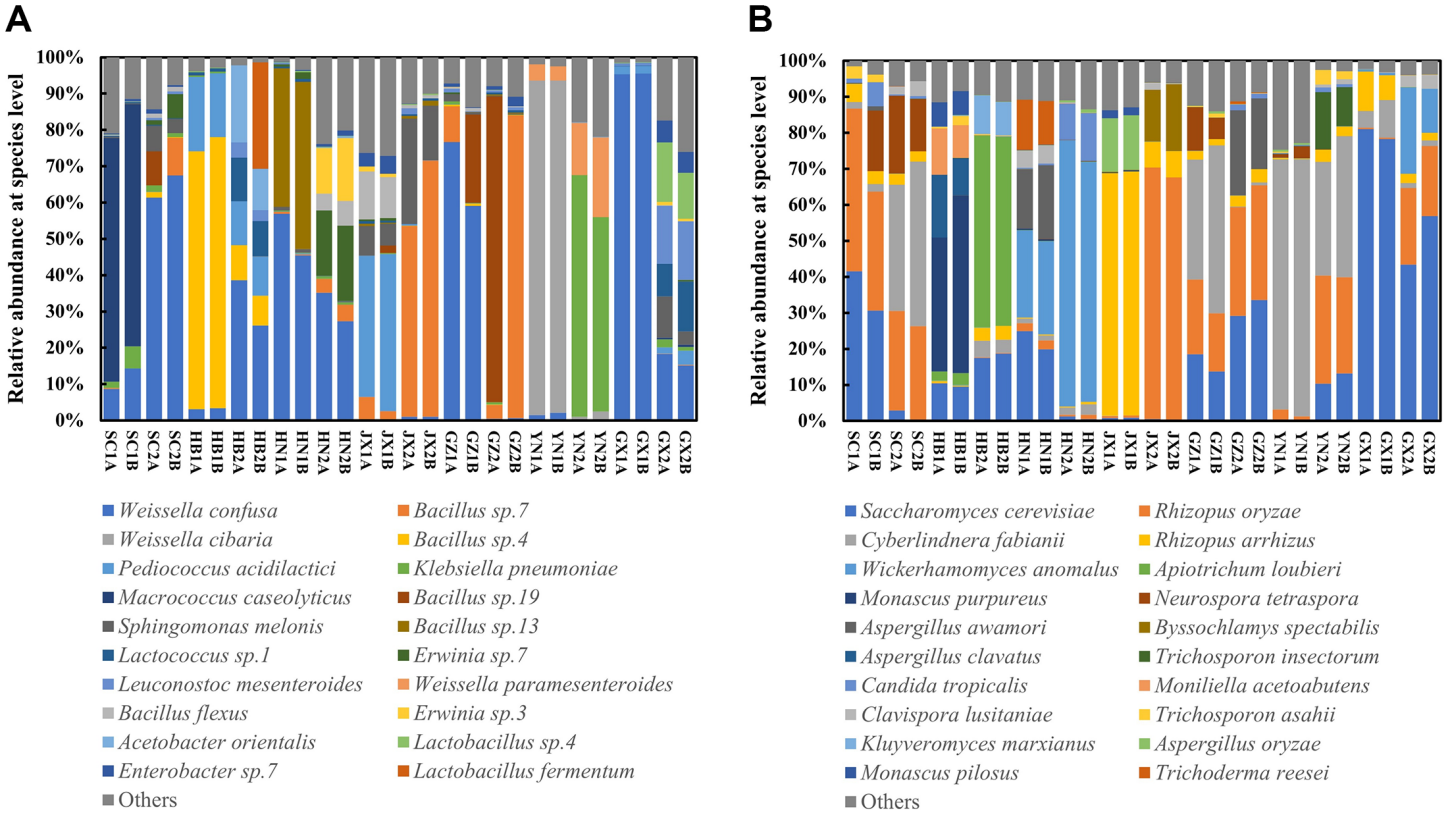
The microbial community of *Xiaoqu* from seven provinces in southern China, including bacterial species level (A) and fungal species level (B).

At the species level, the microbial composition of *Xiaoqu* exhibited greater complexity and variation among samples (Fig. 5). The top 20 species of bacteria are shown in Fig. 5A, the vast majority belonged to LABs and *Bacillus*, consistent with prior study [10]. *Weissella confusa*, *Bacillus* sp.7, *Weissella cibaria*, *Bacillus* sp.4, and *Pediococcus acidilactici* constituted the top 5 bacterial species in abundance (Fig. 5A). Similarly, the top 5 fungal species comprised *Saccharomyces cerevisiae*, *Rhizopus oryzae*, *Cyberlindnera fabianii*, *Rhizopus arrhizus*, and *Wickerhamomyces anomalus* (Fig. 5B). Furthermore, all top 20 fungi species belonged to yeasts and molds, reaffirming their roles in saccharification and alcohol fermentation during baijiu brewing [3, 34]. Although many species from *Xiaoqu* were detected by HTS and plate isolation, there are some differences, especially at the level of bacterial species composition. Notably, *Weissella confusa* and *Weissella cibaria* ranked as the top two abundant LABs via HTS (Fig. 5A), whereas no *Weissella* strains were isolated under cultivation conditions (Fig. 2A). This disparity may stem from the unculturable of these related species of *Weissella* after a long time of dry state. Conversely, fungal isolation results aligned well with HTS data, indicating that all fungal species in *Xiaoqu* samples belonged to yeasts and molds, with *S. cerevisiae* being the most abundant fungus (Figs. 2B and 5B). Consequently, bacterial communities in different *Xiaoqu* samples exhibited significant variation but consistently featured LABs and *Bacillus*, while fungal communities showed less variability and predominantly comprised yeasts and molds. These findings aligned with Zhao’s study regarding the dominance of LABs and *Bacillus* in *Xiaoqu* [16]. However, the observed variation in fungal communities differs from previous reports, possibly due to regional climatic differences.

### Relationships between Microbial Community and Physicochemical Properties & Enzyme Activities

**Fig. 6:**
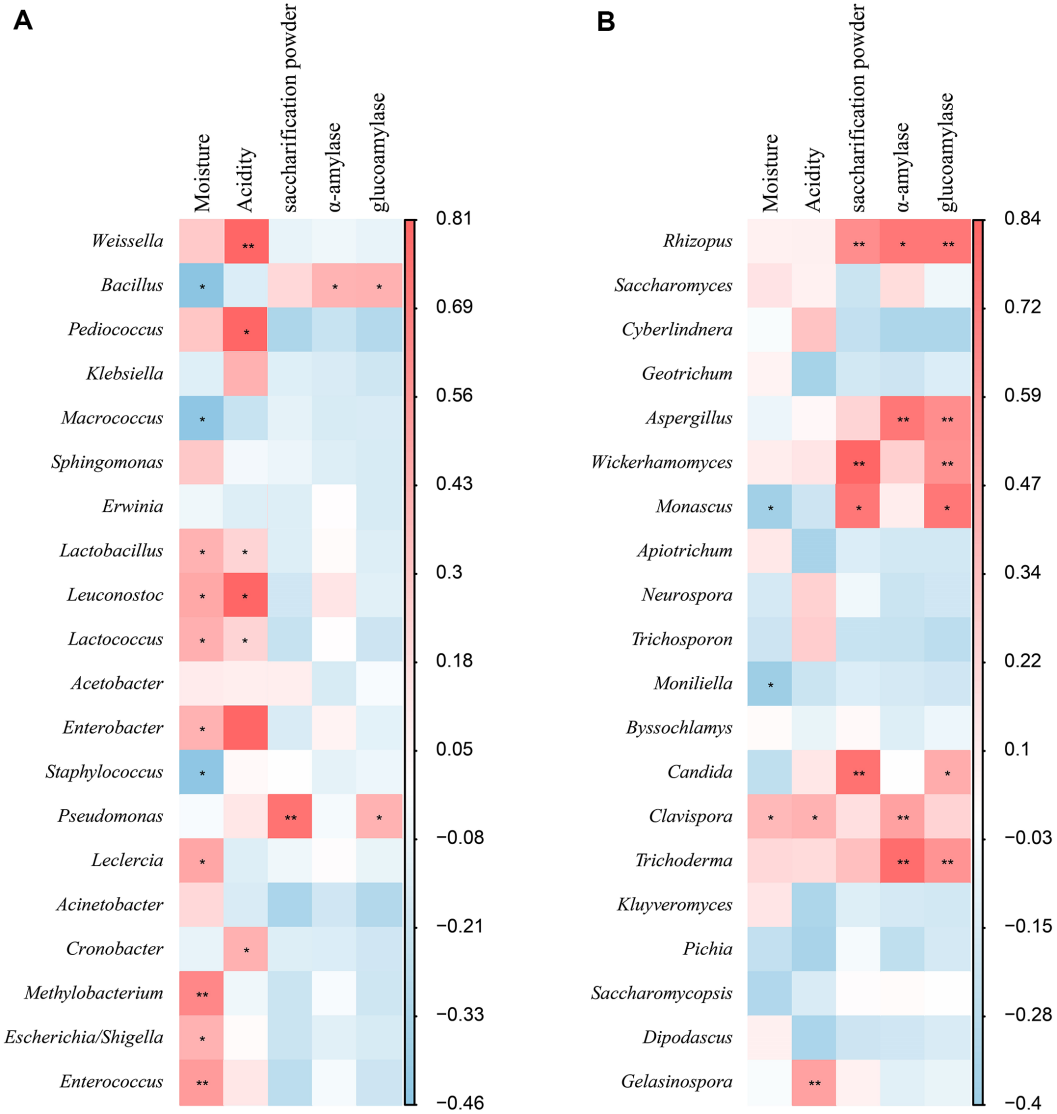
The heatmap of relationships between physicochemical properties & enzyme activities and dominant microbes. The relationships to bacteria were showed in (A) and those to fungi were exhibited in (B). The dominant microbes at genus level (top 20) were selected to analyze the correlations to physicochemical properties and enzyme activities using Pearson correlation coefficient (*p < 0.05, and **p < 0.01). R value was set at 0.6.

The physicochemical properties crucially influence the microbial community composition and succession during *Jiuqu* fermentation, with acidity, temperature, and moisture among the core factors [3, 34]. Concurrently, microbial composition strongly correlates with *Jiuqu* enzyme activities [4]. This study explored the relationship between core microbes at the genus level and physicochemical properties, along with enzyme activities, through Pearson correlation analysis. LABs (*Weissella*, *Pediococus*, *Lactobacillus*, *Leuconostoc*, *Lactococcus*) were positively correlated with acidity (Fig. 6A), consistent with their role in lactic acid production, which elevates acidity levels [10]. Meanwhile, bacteria generally showed no positive correlation with enzyme activities, suggesting their limited contribution to *Xiaoqu* enzyme activities, except for *Bacillus*, which provides a modest amount of α-amylase, glucoamylase, and saccharification power (Fig. 6A) [33]. In contrast, fungi mainly demonstrated a positive correlation with α-amylase, glucoamylase, and saccharification power, including *Rhizopus*, *Aspergillus*, *Monascus*, *Wickerhamomyces*, *Candida*, *Clavispora*, and *Trichoderma* (Fig. 6B). This underscores the importance of molds and certain yeasts in maintaining normal enzyme activities in *Xiaoqu*. Therefore, the core microbial community in *Xiaoqu* appears relatively straightforward: LABs maintain acidity to prevent contamination [5, 15], while *Bacillus*, with its spore-producing ability to withstand dry environments, aids in starch hydrolysis through enzyme production [19, 33]. However, molds and yeasts play the most vital role, with molds possessing robust α-amylase and glucoamylase capabilities to produce reducing sugars from starch, essential for yeast proliferation and ethanol production, thus ensuring fermentation and baijiu quality stability [3].

This study investigated the differences in microbial diversity and enzymes activity of finished *Xiaoqu* from southern China. A total of 22 bacterial species and 17 fungal species were isolated. However, there is a lack of understanding of the microbial community over the time dimension during the *Xiaoqu* fermentation processes. Therefore, future research could further explore the microbiota of *Xiaoqu* on a temporal scale to discover and isolate more functional microbes, providing microbial resources for the production of artificial *Xiaoqu* based on synthetic microbial community in the future. This will not only contribute to a deeper understanding of the brewing processes but also provide a scientific basis for improving the quality of *Xiaoqu* baijiu products and promoting the healthy development of *Xiaoqu* baijiu industry in southern China.

## Conclusion

This study is the first to use high-throughput sequencing to compare *Xiaoqu* samples from multiple provinces, providing a comprehensive overview of microbial diversity and its implications for baijiu production. The comprehensive analyses of *Xiaoqu* reveals significant regional variations in the microbial composition and enzyme activities. Key findings include the predominance of *Bacillus* (*Bacillus subtilis*), LABs, yeasts (*Saccharomyces cerevisiae*), and molds in microbial community, underscoring its pivotal roles in the correlation between microbial diversity and enzyme activities. The positive association of LABs with acidity maintenance and molds with enzyme activities were found. These results have significant implications for optimizing baijiu production. Understanding the microbial composition of *Xiaoqu* can lead to the development of tailored microbial inoculants, enhancing fermentation efficiency and product quality. However, the temporal changes in microbial community were not investigated. The future research could include longitudinal sampling to understand microbial dynamics throughout the fermentation processes.

## Supplemental Materials

Supplementary data for this paper are available on-line only at http://jmb.or.kr.



## Figures and Tables

**Table 1:** The sampling information of *Xiaoqu* from seven provinces in southern China.

Xiaoqu samples	Provinces	Locations
SC1-1/SC1-2	Sichuan	Luzhou city
SC2-1/SC2-2	Sichuan	Penghua city
HB1-1/HB1-2	Hubei	Daye city
HB2-1/HB2-2	Hubei	Huangshi city
HN1-1/HN1-2	Hunan	Yongzhou city
HN2-1/HN2-2	Hunan	Jiangyong city
JX1-1/JX2-2	Jiangxi	Leping city
JX2-1/JX2-2	Jiangxi	Yingtan city
GZ1-1/GZ1-2	Guizhou	Tongren city
GZ2-1/GZ2-2	Guizhou	Kaili city
YN1-1/YN1-2	Yunnan	Kunming city
YN2-1/YN2-2	Yunnan	Kunming city
GX1-1/GX1-2	Guangxi	Hechi city
GX2-1/GX2-2	Guangxi	Guilin city

**Table 2:** The α-diversity of *Xiaoqu* from seven provinces in southern China.

ID	Bacteria			Fungi		
Samples	Chao1	Shannon	Good Coverage	Chao1	Shannon	Good Coverage
SC1	42.10 ± 2.97	1.26 ± 0.04	0.9999 ± 4.4E-05	66.52 ± 19.12	1.65 ± 0.48	1.0000 ± 1.3E-05
SC2	58.71 ± 6.06	1.51 ± 0.21	0.9999 ± 2.1E-05	71.81 ± 0.49	1.66 ± 0.09	0.9998 ± 2.7E-05
HB1	26.88 ± 3.71	0.91 ± 0.04	0.9999 ± 2.1E-05	106.11 ± 4.09	2.19 ± 0.11	0.9998 ± 6.7E-05
HB2	23.50 ± 7.78	1.76 ± 0.09	0.9999 ± 1.0E-03	93.06 ± 0.08	1.92 ± 0.02	0.9997 ± 4.0E-05
HN1	41.25 ± 8.84	1.07 ± 0.14	1.0000 ± 6.2E-05	92.06 ± 3.00	2.08 ± 0.08	0.9999 ± 4.1E-05
HN2	44.50 ± 3.54	2.12 ± 0.01	0.9999 ± 6.2E-05	180.75 ± 27.22	1.45 ± 0.02	0.9995 ± 1.0E-03
JX1	88.38 ± 7.60	2.49 ± 0.05	0.9999 ± 6.1E-05	150.53 ± 5.21	1.42 ± 0.02	0.9997 ± 1.1E-03
JX2	50.50 ± 4.75	1.40 ± 0.26	0.9999 ± 1.2E-03	89.99 ± 0.08	1.49 ± 0.02	0.9998 ± 4.4E-05
GZ1	48.67 ± 5.19	1.17 ± 0.12	0.9999 ± 8.3E-05	121.92 ± 3.74	1.99 ± 0.09	0.9998 ± 1.1E-03
GZ2	58.67 ± 0.47	0.86 ± 0.02	0.9999 ± 4.1E-05	131.59 ± 4.11	2.09 ± 0.06	0.9997 ± 1.1E-03
YN1	21.00 ± 8.49	0.41 ± 0.03	0.9999 ± 4.1E-05	97.33 ± 3.77	1.13 ± 0.01	0.9997 ± 4.0E-05
YN2	44.75 ± 5.30	1.45 ± 0.21	1.0000 ± 2.7E-05	94.94 ± 15.94	1.98 ± 0.14	0.9997 ± 6.7E-05
GX1	9.00 ± 0.01	0.26 ± 0.01	1.0000 ± 1.2E-05	26.71 ± 2.42	0.86 ± 0.03	0.9999 ± 5.2E-05
GX2	67.00 ± 0.03	2.65 ± 0.19	0.9999 ± 1.0E-03	44.31 ± 3.91	1.32 ± 0.09	0.9999 ± 2.7E-05
