# Supplementary material for: High-Throughput Sequencing Analysis of Microbiota and Enzyme Activities in Xiaoqu from Seven Provinces in Southern China
Source: J Microbiol Biotechnol. 2024 Sep 9;34(11):2290–300. doi: 10.4014/jmb.2405.05029 (PMC11637830; doi:10.4014/jmb.2405.05029)
Supplement: Supplementary file 1 [file jmb-34-11-2290-supple.pdf]

J. Microbiol. Biotechnol. 2024. 34(11): 2290–2300
https://doi.org/10.4014/jmb.2405.05029

Supplementary Figures

Interpreting the microbiota and enzyme activities of *Xiaoqu* from seven provinces in southern China

Weiwei Dong[1, 2†], Jingjing Zhang[1†], Menglin Zou[2], Liang Chen[1], Liping Zhu[1], Long Zhang[1], Gang Zhang[1], Jie Tang[1], Qiang Yang[1], Yuanliang Hu[1, 2*], Shenxi Chen[1, 2*]

[1] Hubei key Laboratory of Quality and Safety of Traditional Chinese Medicine Health Food, Jing Brand Co., Ltd., Daye, Hubei 435100, China.
[2] Hubei Key Laboratory of Edible Wild Plants Conservation and Utilization, College of Life Sciences, Hubei Normal University, Huangshi 435002, China.

[†] These authors contributed equally to this work

***Corresponding authors:**

Yuanliang Hu, E-mail address: ylhu@hbnu.edu.cn,

Shenxi Chen, E-mail address: chenshenxi2006@163.com,

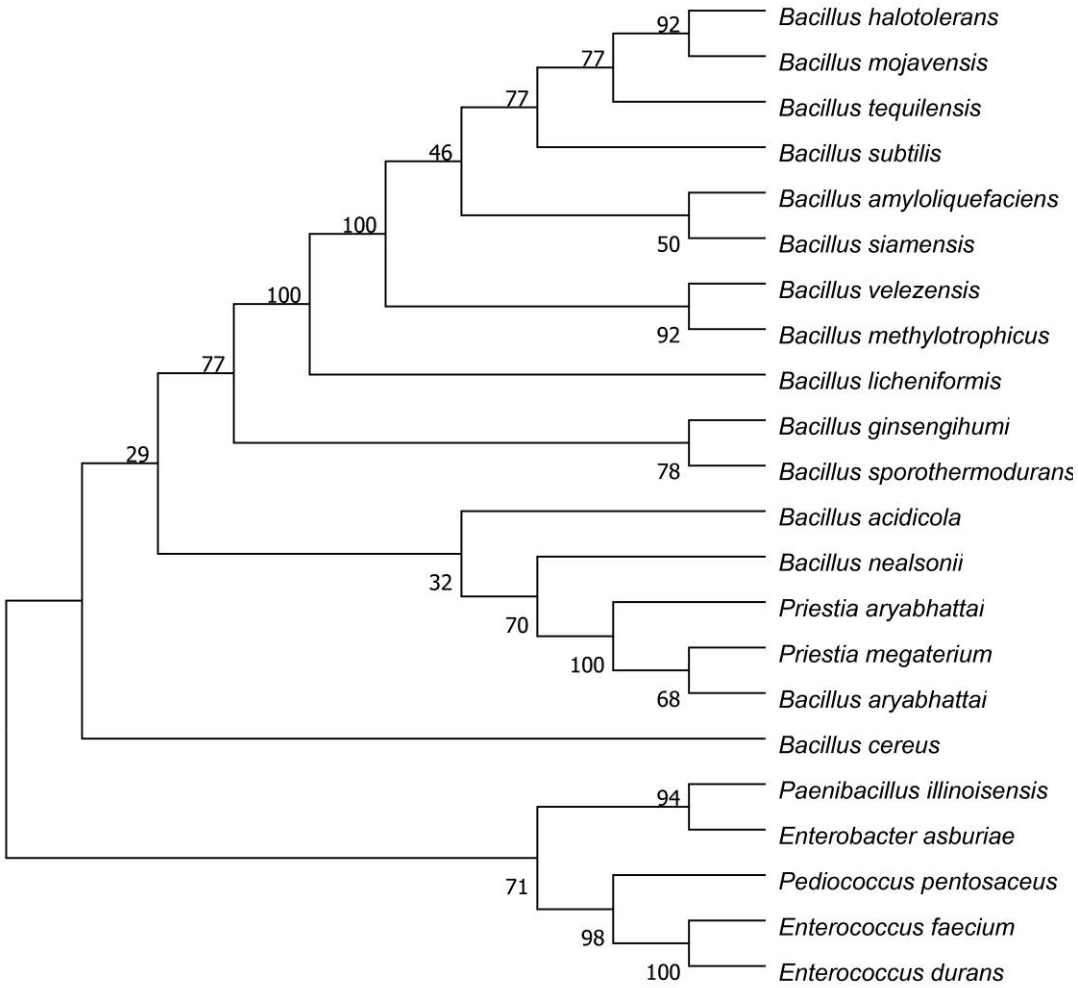

Fig. S1. The phylogenetic tree (neighbor-joining) of the isolated 22 bacterial species (numbers at the nodes are the bootstrap values from 500 replicates).

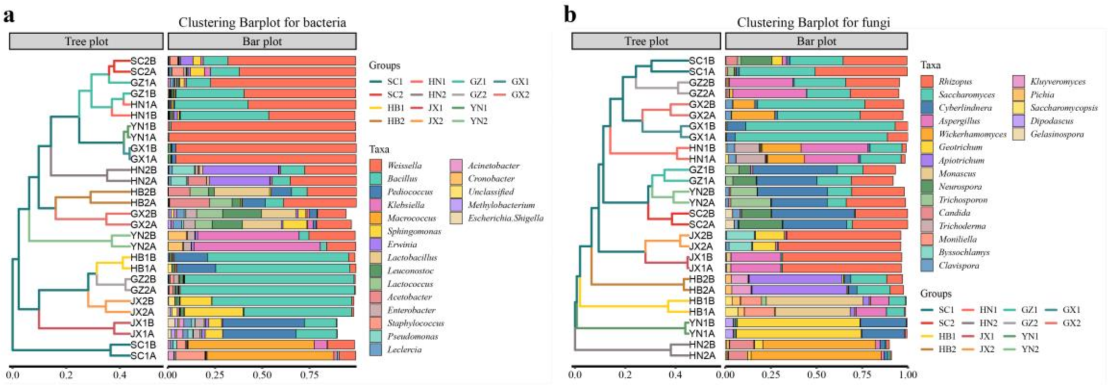

Fig. S2. Clustering analysis based on microbial compositions at genus level among all *Xiaoqu* samples from seven provinces in southern China, including bacteria (a) and fungi (b).
